# Substance use patterns among a global sample of transgender and non-binary people during the COVID-19 pandemic

**DOI:** 10.1186/s44263-023-00014-5

**Published:** 2023-09-18

**Authors:** Henri M. Garrison-Desany, Chase P. Childress, Nicole McConico, Brooke A. Jarrett, Sean Howell, Jennifer L. Glick

**Affiliations:** 1 Department of Epidemiology, Johns Hopkins Bloomberg School of Public Health, 615 N. Wolfe Street, Baltimore, MD 21205 USA; 2 School of Criminology and Criminal Justice, Northeastern University, Boston, MA USA; 3 Department of Mental Health, Johns Hopkins Bloomberg School of Public Health, Baltimore, MD USA; 4 Hornet, San Francisco, CA USA; 5 Department of Health, Behavior, and Society, Johns Hopkins Bloomberg School of Public Health, Baltimore, MD USA

**Keywords:** Transgender, Non-binary, LGBT, COVID-19, Substance use

## Abstract

**Background:**

Transgender and non-binary (TNB) people are at high risk of substance misuse compared to cisgender individuals. Few studies have described substance use among non-binary people, and many studies have focused solely on samples from Western countries. In this global study of TNB people, we sought to identify intra-group differences, risk factors, and COVID-related changes in the use of tobacco, alcohol, and cannabis.

**Methods:**

We used cross-sectional data from 926 TNB users of the Hornet app across 76 countries between October and November 2020. Participants self-reported the use of tobacco, alcohol, and cannabis in the past 6 months and any changes in use during the pandemic. We generated descriptive statistics and used logistic regression to assess substance use between TNB subgroups, identify risk factors for each substance by gender identity, and identify changes in substance use before and during the pandemic.

**Results:**

Most TNB participants used tobacco (53.7%, *n* = 498) and alcohol (66.3%, *n* = 614). Non-binary participants had increased odds of using cannabis (adjusted odds ratio: 1.62, 95% CI 1.03, 2.55) compared to transfeminine participants. Participants’ geographic region of residence was most associated with higher substance use, compared to other potential factors. Most participants reported increases in at least one substance during COVID-19 (54.2%, *n* = 276 of 518 responses).

**Conclusions:**

In this global TNB sample, we found that substance use varied by gender identity, and changes in substance use during the pandemic varied by TNB sub-groups. We join researchers calling for gender-specific tailoring of substance-related services for TNB clients and urge further studies with greater inclusion and disaggregation of non-binary and transmasculine individuals to support better-informed analysis of transgender health.

**Supplementary Information:**

The online version contains supplementary material available at 10.1186/s44263-023-00014-5.

## Background

Substance use is a global public health crisis. If current trends persist, tobacco is predicted to lead to the deaths of a cumulative one billion people worldwide by the year 2100 [1]. Alcohol contributes to nearly three million premature deaths annually [2], and nearly 200 million individuals were estimated to have used cannabis in 2019 [3]. Tobacco, alcohol, and cannabis are the three most common substances used in the world [4]. Substance use is motivated by a number of complex and interrelated reasons, with both positive and detrimental outcomes, including as a coping mechanism but also other perceived benefits like increased socialization. Chronic substance use is associated with a myriad of health problems including risk of overdose, attention and memory issues, cirrhosis of the liver associated with alcohol use, and lung cancer associated with tobacco use [5–7]. The costs of substance misuse also include economic loss, academic disruption, and criminal justice involvement [6]. Therefore, understanding the risks for initiation and for changes in substance use behaviors is important to support individuals seeking to reduce or cease their use.

The coronavirus (COVID-19) pandemic, and related precautions, exacerbated socio-environmental circumstances associated with substance use for many people, as quarantines and closures resulted in a decline in available social support and ability to cope with stressors [8–11]. Attention to substance use during the pandemic is critical because individuals engaged in substance use are at increased risk for, among other things, heightened COVID-19 severity, possibly due to chronic medical conditions and reduced access to healthcare [12]. Substance use research during the COVID-19 pandemic is limited in geographic scope, as many studies were conducted in high-income nations with few low- to middle-income countries included [13].

However, some evidence suggests that COVID-19 has altered the pattern of use and choice of drugs, including increased use of legal drugs including alcohol [14], increased cannabis use, and increased use of other substances (including stimulants, narcotics, etc.) [15, 16]. One study estimated 13.3% of adults in the USA began or increased their use of at least one substance in 2020 [17].

There are numerous gaps in research on transgender people’s substance use since the COVID-19 pandemic. Research prior to the COVID-19 pandemic suggests that transgender and non-binary (TNB) people have a higher prevalence of substance use compared to other minority populations, such as cisgender sexual minority people; however, the distinction between transgender groups is rarely investigated [18–21]. Additionally, while five studies to date have reported on differences in substance use between transgender women and men, none has reported substance use rates for non-binary participants [21–27]. The vast majority of transgender substance use studies have concentrated primarily in the USA and Canada and occur among samples of transgender women at increased risk of HIV [22]. This has led to a substantial gap in literature on the substance use behaviors and risk factors for non-binary people, transmasculine people, and transgender communities outside of North America. A lack of data disaggregated by subgroups of gender identity may lead to inaccurately generalizing findings based on transgender women, as well as foregone opportunities to better understand health and disease processes, such as the role of gender in substance use through comparative analysis [28]. The exclusion of non-binary and transmasculine individuals from research also reinforces their exclusion from programmatic social supports, gender-affirming services, and policy processes [29, 30].

Minority stress theory [31] suggests that TNB people likely experience increased socio-environmental stressors that can lead to substance use as a coping mechanism [32, 33]. Both external and internal stressors, such as violence and internalized stigma, may induce psychological distress and lead to substance use [33–35]. Experiencing violence and stigma are associated with increased use of tobacco, alcohol, and non-medical prescription drugs among TNB populations [22, 36–39], giving credence to this theory. However, stigmas and gender norms affect separate sub-groups of TNB people differently due to varied experiences of gender identity and expression, cultural norms, health systems, socio-economic realities, and geographic location [40, 41]. Therefore, minority stress theory would posit that subgroups of non-binary and transgender people defined by gender experience and with different backgrounds experience differential risks of and relationships to substance use based on their respective minority stress. However, this hypothesis has rarely been quantitatively investigated in literature.

TNB populations may have been particularly vulnerable to pandemic-related stressors due to their pre-existing economic precarity, reduced healthcare access, and discrimination when accessing healthcare services [42–46]. However, the exclusion of transgender people from the general substance use literature leaves unclear the degree to which transgender status influences substance use, and how this may differ by gender identity group [28]. The primary goal of this paper is to investigate intra-group differences between transgender and non-binary participants in a global sample. We present findings identifying specific risk factors to better inform interventions tailored to specific TNB communities and reduce substance use disorders among these vulnerable populations.

## Methods

### Data collection

We used cross-sectional data collected via self-report between October 25 and November 26, 2020, from the Global COVID-19 Disparities Survey, an online sample of users on the Hornet dating and social networking application (“Hornet app”). Users of the Hornet app were considered eligible for the study if they were 18 years or older and provided electronic informed consent. The study was available in ten languages: English, Turkish, Arabic, Russian, Spanish, French, Simplified and Traditional Chinese, Thai, and Malay. Translations were conducted by members within the broader research team and partners within countries who spoke the respective languages. All questions, including gender identity and sexuality questions, were reviewed by members who spoke the respective languages and were themselves transgender, non-binary, and/or queer. Notably, our study was therefore limited to participants who spoke these languages, for whom the app was available (e.g., not banned or limited in their country’s respective app stores), and who had access to a smartphone and the app itself. Recruitment was conducted via message directly to a user’s Hornet inbox with a link to a Qualtrics survey to assess eligibility.

### Gender identity definition

We defined gender identity using two questions: sex at birth (“male,” “female,” or “intersex or diagnosed with a difference of sex development”) and gender, for which participants could select more than one response (“gender non-binary/gender diverse [also genderqueer, gender nonconforming, gender expansive],” “man,” “woman,” “transgender man”, “transgender woman,” “agender,” “I don’t know,” and “I cannot or do not wish to answer this question”). We recorded gender identity into three categories: (1) “non-binary,” including non-binary and agender regardless of sex assigned at birth; (2) “transmasculine,” including participants assigned female or intersex at birth who identified as “man” or “transgender man;” and (3) “transfeminine,” including participants assigned male or intersex at birth who identified as “woman” or “transgender woman.”

### Outcomes

The three main outcomes are recent substance use, defined as use within the 6 months preceding survey completion, across (1) tobacco, (2) alcohol, and (3) cannabis, each binarized (any/none). Participants responded to: “In the past six months, have you used X?” Individuals who responded “no” or “I don’t know” were binarized as “no,” leading to a more conservative prevalence estimate than had they been grouped with “yes” categories, given assumptions that an “I don’t know” response could indicate contamination or an unexpected effect of a substance, neither which are possible to confirm in our study design. As a secondary analysis, we analyzed responses to change in use since COVID-19 began. Participants who reported any substance use were also asked, “How has your X use changed since the COVID-19 crisis began?” with response options: “decreased,” “no change,” and “increased.”

### Analytic sample

We included TNB participants who responded to three substance use questions, i.e., tobacco, alcohol, and cannabis use in the past 6 months (Additional file 1: Fig. S1).

### Covariates and exposures

We created a directed acyclic graph (DAG, Additional file 1: Fig. S2) based on a literature search of factors associated with substance use in TNB samples: [12, 22, 25, 33, 47]. We evaluated socioeconomic status, age, education, gender-based health service discrimination, gender-based discrimination from police, urban or rural residence, disability status, and job loss via self-report. Socioeconomic status was divided into self-identification as lower, lower-middle, upper-middle, and upper class. Education was categorized as not finishing secondary school or trade school, finishing secondary school or trade school, and attending some higher education or finishing higher education. Age was categorized according to the interquartile range (IQR) of < 25 years, 25 to 40 years, and > 40 years of age. Urbanicity/rurality was defined as three categories: living in a rural area, a small city (including suburbs near large cities), or a large city. The following were addressed as binary variables: disability (i.e., “Do you have a physical or mental condition that limits your movements, senses, or daily activities?”) job loss due to COVID-19, discrimination when seeking police services, and discrimination when seeking health services (i.e., “Have you ever been refused [“health” or “police”] services at any point because of your gender identity or expression, sex characteristics, or sexual orientation?”). We assessed global region of residence based on self-reported country of residence, then categorized responses according to the World Health Organization (WHO) regional defined as European (EUR), American (AMR), Southeast Asia (SEAR), Eastern Mediterranean (EMR), Western Pacific (WPR), and African (AFR) regions [48].

### Missingness

Missingness was assessed across all variables by testing for association between substance use and missing a given variable and was determined to be missing at random. Multiple imputation via chained equation was used across 50 datasets for 30 iterations each. All models were fit independently in each dataset and estimates were pooled using Rubin’s rules [49].

### Statistical analysis

First, we generated descriptive statistics of our sample, stratified by TNB sub-group (i.e., non-binary, transmasculine, transfeminine). To estimate the association of each covariate with each substance use variable, we used pooled estimates from logistic regression models across multiple imputed datasets, stratified by gender. Finally, we generated descriptive statistics of changes in substance use during the COVID-19 pandemic. We determined statistical significance using a *p*value threshold of 0.05, generated 95% confidence intervals (95% CI), and conducted analyses in Stata 17 [50]. To pool multiple imputation estimates for predicted proportions, we used the mimrgns package in Stata [51].

## Results

### Descriptive statistics

Our final analytic sample included 926 participants who were identified as non-binary (*n* = 620, 67.0%), transfeminine (*n* = 231, 25.0%), or transmasculine (*n* = 75, 8.1%; Table 1). Most participants had either smoked tobacco (53.8%, *n* = 498) or drank alcohol (64.5%, *n* = 614) in the six months prior to the survey. The plurality of participants had both smoked tobacco and drank alcohol (40.8%, *n* = 368). However, only a minority (14.5%, *n* = 135) had consumed cannabis.

**Table 1:** Descriptive statistics of transgender participants, by gender identity among transgender and non-binary participants

	Non-binary *n* = 620 (67.0%)	Transfeminine *n* = 231 (25.0%)	Transmasculine *n* = 75 (8.1%)	*P* value
Tobacco				0.38
No	273 (44.0%)	89 (38.5%)	30 (40.0%)	
Yes	327 (52.7%)	133 (57.6%)	38 (50.7%)	
Missing	20 (3.2%)	9 (3.9%)	7 (9.3%)	
Alcohol				0.04
No	192 (31.0%)	81 (35.1%)	15 (20.0%)	
Yes	411 (66.3%)	144 (62.3%)	59 (78.7%)	
Missing	17 (2.7%)	6 (2.6%)	1 (1.3%)	
Cannabis				0.36
No	470 (75.8%)	185 (80.1%)	56 (74.7%)	
Yes	97 (15.6%)	28 (12.1%)	9 (12.0%)	
Missing	53 (8.5%)	18 (7.8%)	10 (13.3%)	
Socioeconomic status				0.03
Lower	78 (12.6%)	31 (13.4%)	14 (18.7%)	
Lower middle	252 (40.7%)	75 (32.5%)	19 (25.3%)	
Upper middle	171 (27.6%)	64 (27.7%)	25 (33.3%)	
Upper	32 (5.2%)	22 (9.5%)	4 (5.3%)	
Missing	87 (14.0%)	39 (16.9%)	13 (17.3%)	
Education				0.11
Did not complete secondary school	30 (4.8%)	11 (4.8%)	8 (10.7%)	
Completed secondary or trade school	154 (24.8%)	65 (28.1%)	21 (28.0%)	
Attended or completed higher education	355 (57.3%)	120 (52.0%)	34 (45.3%)	
Missing	81 (13.1%)	35 (15.2%)	12 (16.0%)	
Age category				0.003
≤ 25	173 (27.9%)	68 (29.4%)	20 (26.7%)	
25–40	310 (50.0%)	123 (53.3%)	25 (33.3%)	
> 40	137 (22.1%)	40 (17.3%)	30 (40.0%)	
Missing	0	0	0	
Refused health services				0.03
No	263 (42.4%)	84 (36.4%)	28 (37.3%)	
Yes	95 (15.3%)	49 (21.2%)	6 (8.0%)	
Missing	262 (42.3%)	98 (42.4%)	41 (54.7%)	
Refused police services				0.16
No	220 (35.5%)	72 (31.2%)	23 (30.7%)	
Yes	110 (17.7%)	54 (23.4%)	12 (16.0%)	
Missing	290 (46.8%)	105 (45.5%)	40 (53.3%)	
Disability status				0.82
No	427 (68.9%)	156 (67.5%)	48 (64.0%)	
Yes	58 (9.4%)	24 (10.4%)	8 (10.7%)	
Missing	135 (21.8%)	51 (22.1%)	19 (25.3%)	
Lost job due to COVID-19				0.67
No	424 (68.4%)	156 (67.5%)	45 (60.0%)	
Yes	46 (7.4%)	16 (6.9%)	7 (9.3%)	
Missing	150 (24.2%)	59 (25.5%)	23 (30.7%)	
Rurality/urbanicity				0.02
Rural	45 (6.9%)	11 (4.8%)	3 (4.0%)	
Small city	140 (22.6%)	39 (16.9%)	7 (9.3%)	
Large city	353 (56.9%)	145 (62.8%)	53 (70.7%)	
Missing	84 (13.6%)	36 (15.6%)	12 (16.0%)	
Region				< 0.001
European	312 (50.3%)	142 (61.5%)	40 (53.3%)	
American	67 (10.8%)	22 (9.5%)	12 (16.0%)	
South East Asian	159 (25.7%)	14 (6.1%)	9 (12.0%)	
Eastern Mediterranean	30 (4.8%)	28 (12.1%)	4 (5.3%)	
Western Pacific	17 (2.7%)	6 (2.6%)	4 (5.3%)	
African	3 (0.5%)	5 (2.2%)	1 (1.3%)	
Missing	32 (5.2%)	14 (6.1%)	5 (5.7%)	

“Missing” represents values missing for corresponding variables before multiple imputation by chained equation (MICE). MICE was conducted using all presented covariates as predictors for the missing values

*P* values are reported using ${\chi }^{2}$ tests for categories with ≥ 5 entries per cell, and Fisher’s exact test for categories with < 5 entries per cell

*OR* odds ratio, *CI* confidence interval, *COVID*-*19* novel coronavirus disease 2019, *EUR* European Region, *AMR* Americas Region, *SEAR* Southeast Asian Region, *EMR* Eastern Mediterranean Region, *WPR* Western Pacific Region, *AFR* African Region

Most participants identified themselves as lower-middle class (44.0%, *n* = 346) and attended or finished higher education (55.0%, *n* = 509). More transmasculine people self-identified their socioeconomic status as low (18.7%, *n* = 14) compared to non-binary (12.6%, *n* = 78) or transfeminine people (13.4%, *n* = 31). Most participants were from the EUR region (53.3%, *n* = 494). The second most common regions of residence across each gender identity strata were SEAR for non-binary people (25.7%, *n* = 159), EMR for transfeminine people (12.1%, *n* = 28), and AMR for transmasculine people (16.0%, *n* = 12). The median age was 31 (IQR: 25 to 40) for non-binary participants, 30 (IQR: 25, 38) for transfeminine participants, and 36 (IQR: 25 to 45) for transmasculine participants. Most participants lived in urban areas (59.5%, *n* = 551).

### Substance use differences among TNB people across gender identities

Transmasculine participants were more likely to report recent alcohol use than transfeminine participants (OR 2.04, 95% CI 1.08, 3.78), though this effect was not significant after adjustment (Table 2). Non-binary participants were more likely to report recent cannabis use compared to transfeminine participants (aOR: 1.62, 95% CI 1.03, 2.55). No differences were found between gender identity groups regarding tobacco or cannabis use.

**Table 2:** Differences in substance use risk among transgender participants, by gender identity among transgender and non-binary participants

	Tobacco use in past 6 months			Alcohol use in past 6 months			Cannabis use in past 6 months		
Gender identity	Unadjusted OR (95% CI)	Adjusted OR (95% CI)	Predicted proportion (95% CI)	Unadjusted OR (95% CI)	Adjusted OR (95% CI)	Predicted proportion (95% CI)	Unadjusted OR (95% CI)	Adjusted OR (95% CI)	Predicted proportion (95% CI)
Transfeminine	Ref	Ref	56.39 (49.55, 62.24)	Ref	Ref	64.11 (57.46, 70.76)	Ref	Ref	15.65 (10.78, 20.51)
Non-binary	0.84 (0.61, 1.17)	1.00 (0.71, 1.41)	57.98 (53.94, 62.03)	1.18 (0.84, 1.64)	1.40 (0.97, 2.01)	70.41 (66.67, 74.15)	1.43 (0.95, 2.17)	1.63 (0.98, 2.70)	**22.65 (19.15, 26.15)**
Transmasculine	0.97 (0.56, 1.67)	1.07 (0.58, 1.97)	**62.13 (50.61, 73.66)**	**2.04 (1.08, 3.84)**	**2.12 (1.13, 4.35)**	76.56 (66.41, 86.71)	1.47 (0.76, 2.85)	1.63 (0.52, 3.01)	22.35 (12.74, 31.97)
Socioeconomic status									
Lower middle		1.98 (0.87, 2.21)			1.07 (0.66, 1.74)			1.06 (0.53, 2.15)	
Upper middle		1.30 (0.80, 2.11)			1.10 (0.65, 1.85)			1.04 (0.52, 2.11)	
Upper		1.11 (0.55, 2.27)			1.04 (0.50, 2.15)			1.06 (0.40, 2.82)	
Education									
HS or trade		0.58 (0.28, 1.18)			1.20 (0.56, 2.57)			0.63 (0.23, 1.70)	
College		0.42 (0.20, 0.85)			2.00 (0.98, 4.07)			0.84 (0.32, 2.20)	
Age									
25–40 years		0.92 (0.65, 1.30)			1.09 (0.76, 1.57)			1.57 (0.96, 2.56)	
40 +		0.70 (0.46, 1.06)			0.70 (0.45, 1.08)			0.95 (0.51, 1.77)	
Refused									
Health services		1.07 (0.68, 1.69)			1.04 (0.65, 1.66)			1.46 (0.80, 2.64)	
Police services		1.05 (0.68, 1.64)			0.92 (0.57, 1.47)			1.16 (0.65, 2.04)	
Disability status		1.35 (0.83, 2.19)			1.23 (0.74, 2.03)			**1.96 (1.09, 3.51)**	
Job lost due to COVID		0.81 (0.47, 1.38)			0.98 (0.50, 1.50)			0.97 (0.47, 1.97)	
City									
Small city		0.80 (0.42, 1.51)			1.88 (0.96, 3.67)			1.48 (0.46, 4.75)	
Large city		1.19 (0.65, 2.18)			**1.91 (1.05, 3.48)**			1.78 (0.60, 5.30)	
Region									
American		0.57 (0.36, 0.90)			**2.33 (1.27, 4.30)**			**4.27 (2.51, 7.28)**	
South East Asian		0.41 (0.28, 0.60)			0.45 (0.30, 0.68)			0.71 (0.38, 1.30)	
Eastern Mediterranean		0.99 (0.55, 1.78)			0.50 (0.29, 0.88)			**2.13 (1.00, 4.52)**	
Western Pacific		0.49 (0.22, 1.10)			0.95 (0.39, 2.29)			1.55 (0.54, 4.46)	
African		0.22 (0.04, 1.22)			1.21 (0.23, 6.37)			1.24 (0.13, 11.65)	

Statistically significant results in bold

Models were adjusted for WHO region, urbanicity, socioeconomic status, education, age category, disability status, whether they have refused health services, whether they have been refused police services, and whether they lost their job due to COVID-19

*OR* odds ratio, *CI* confidence interval, *COVID-19* novel coronavirus disease 2019

### Risk factors for substance use by gender identity category

Non-binary participants who had not finished secondary school were more likely to use tobacco recently compared to those who had finished or who had attended college (Table 3). There was no significant association between education and tobacco use among transfeminine or transmasculine participants.

**Table 3:** Differences in factors associated with tobacco, alcohol, and cannabis use in the past six months, stratified by gender identity among transgender and non-binary participants

	**Tobacco**			**Alcohol**			**Cannabis**		
	Non-binary OR (95% CI)	Transfeminine OR (95% CI)	Transmasculine OR (95% CI)	Non-binary OR (95% CI)	Transfeminine OR (95% CI)	Transmasculine OR (95% CI)	Non-binary OR (95% CI)	Transfeminine OR (95% CI)	Transmasculine OR (95% CI)
Socioeconomic status									
Lower middle	1.25 (0.69, 2.25)	1.94 (0.72, 5.22)	0.51 (0.05, 4.88)	0.87 (0.47, 1.60)	1.34 (0.43, 4.20)	2.38 (0.26, 22.13)	1.06 (0.50, 2.26)	1.15 (0.31, 4.31)	5.49 (0.20, 153.81)
Upper middle	1.24 (0.68, 2.25)	2.09 (0.72, 6.06)	0.76 (0.09, 6.48)	0.95 (0.48, 1.87)	1.34 (0.41, 4.37)	2.69 (0.15, 47.07)	1.03 (0.46, 2.31)	1.48 (0.36, 6.12)	1.37 (0.05, 37.15)
Upper	1.18 (0.46, 3.01)	1.49 (0.41, 5.39)	0.13 (0.004, 3.99)	1.53 (0.47, 4.97)	1.04 (0.24, 4.63)	0.33 (0.01, 10.13)	0.91 (0.27, 3.06)	0.91 (0.15, 5.46)	0.92 (0.01, 81.29)
Education									
HS or trade	**0.34 (0.11, 0.99)**	3.27 (0.61, 17.40)	0.26 (0.01, 8.17)	0.83 (0.33, 2.11)	1.18 (0.22, 6.36)	0.26 (0.01, 9.07)	0.63 (0.20, 1.94)	0.38 (0.05, 2.80)	0.16 (0.004, 6.17)
College	**0.28 (0.10, 0.82)**	1.76 (0.35, 8.81)	0.16 (0.1, 5.48)	1.40 (0.57, 3.48)	2.89 (0.59, 14.09)	0.51 (0.02, 15.07)	0.84 (0.29, 2.50)	0.55 (0.09, 3.43)	0.83 (0.04, 18.33)
Age									
25–40 years	0.87 (0.57, 1.33)	0.94 (0.45, 1.96)	0.44 (0.06, 3.13)	0.92 (0.47, 1.48)	1.17 (0.54, 2.53)	2.05 (0.19, 21.88)	1.52 (0.90, 2.58)	0.98 (0.38, 2.52)	4.13 (0.28, 61.25)
40 +	0.77 (0.46, 1.28)	0.42 (0.16, 1.07)	0.61 (0.09, 4.08)	**0.54 (0.31, 0.93)**	0.59 (0.21, 1.61)	2.64 (0.32, 22.08)	1.05 (0.54, 2.02)	0.45 (0.11, 1.75)	2.33 (0.06, 88.41)
Refused									
Health services	0.86 (0.49, 1.51)	1.39 (0.49, 3.96)	2.03 (0.07, 59.94)	0.81 (0.41, 1.59)	1.25 (0.40, 3.89)	1.53 (0.06, 38.18)	1.43 (0.67, 3.04)	1.78 (0.55, 5.77)	1.01 (0.02, 42.86)
Police services	1.04 (0.60, 1.78)	1.24 (0.49, 3.14)	0.67 (0.04, 11.15)	0.91 (0.49, 1.68)	1.40 (0.54, 3.64)	0.91 (0.05, 16.52)	1.10 (0.50, 2.40)	0.67 (0.21, 2.07)	2.37 (0.10, 55.40)
Disability status	1.04 (0.56, 1.95)	1.99 (0.59, 6.80)	1.24 (0.12, 12.80)	1.06 (0.45, 2.49)	1.20 (0.37, 3.94)	0.76 (0.03, 18.84)	1.76 (0.80, 3.89)	2.88 (0.83, 9.99)	3.15 (0.12, 79.82)
Job lost due to COVID	0.75 (0.40, 1.42)	0.86 (0.25, 2.99)	0.53 (0.05, 5.82)	1.28 (0.60, 2.74)	0.62 (0.16, 2.38)	0.23 (0.01, 4.69)	1.12 (0.50, 2.53)	0.49 (0.09, 2.75)	0.96 (0.03, 28.63)
City									
Small city	0.87 (0.40, 1.89)	0.38 (0.06, 2.20)	0.55 (0.004, 71.40)	1.66 (0.76, 3.62)	11.71 (1.32, 104.06)	Omitted	0.73 (0.25, 2.17)	0.81 (0.11, 6.01)	Omitted
Large city	1.15 (0.55, 2.38)	0.95 (0.19, 4.81)	0.41 (0.004, 42.09)	1.85 (0.88, 3.89)	3.92 (0.57, 26.72)		1.32 (0.49, 3.60)	0.45 (0.07, 3.15)	
Region									
American	0.70 (0.40, 1.23)	0.65 (0.23, 1.84)	**0.08 (0.01, 0.74)**	2.81 (1.26, 6.26)	2.46 (0.63, 9.61)	0.51 (0.06, 4.11)	**4.58 (2.50, 8.43)**	2.33 (0.71, 7.67)	NA
South East Asian	**0.38 (0.25, 0.59)**	0.36 (0.10, 1.33)	1.85 (0.20, 16.78)	**0.49 (0.31, 0.77)**	0.21 (0.05, 0.88)	0.24 (0.03, 2.07)	0.80 (0.45, 1.45)	0.20 (0.02, 2.11)	3.85 (0.37, 40.00)
Eastern Mediterranean	1.92 (0.73, 5.01)	0.66 (0.25, 1.72)	2.28 (0.10, 51.25)	0.64 (0.27, 1.53)	0.38 (0.14, 1.01)	NA	**2.67 (1.10, 6.49)**	0.89 (0.26, 3.11)	9.44 (0.33, 272.96)
Western Pacific	0.36 (0.13, 1.01)	0.20 (0.03, 1.37)	NA	0.75 (0.26, 2.18)	1.26 (0.15, 10.76)	NA	1.42 (0.43, 4.71)	NA	0.49 (0.01, 23.68)
African	0.30 (0.03, 3.61)	0.15 (0.01, 2.07)	NA	NA	1.70 (0.15, 19.86)	NA	2.67 (0.21, 34.23)	1.14 (0.07, 18.74)	NA

Statistically significant results in bold (*P* < 0.05)

NA” refers to estimates that could not be generated due to no participants reporting the factor of interest

“Omitted” refers to estimates that could not be generated due to too few participants (e.g., 4 transmasculine participants were in rural areas) such that the pooled estimates of the multiply imputed data did not converge when this variable was included. Therefore, it was removed from the assessed model

Of our participants, 97 non-binary people, 28 transfeminine people, and 9 transmasculine people reported cannabis use in the last 6 months and were coded as “yes” for our binary outcome

*OR* odds ratio, *CI* confidence interval, *COVID-19* novel coronavirus disease 2019, *EUR* European Region, *AMR* Americas Region, *SEAR* Southeast Asian Region, *EMR* Eastern Mediterranean Region, *WPR* Western Pacific Region, *AFR* African Region

Non-binary participants over 40 years of age were less likely to have consumed alcohol recently than younger (< 25 years) non-binary participants (OR 0.54, 95% CI 0.31, 0.93). Additionally, non-binary participants from the SEAR region reported reduced alcohol use compared to the EUR region (OR 0.49, 95% CI 0.31, 0.77).

Region of residence was the most predictive factor for cannabis use among non-binary participants. Non-binary participants in the AMR region had 4.58 (95% CI 2.50, 8.43) times the odds of cannabis use compared to the EUR region. Additionally, the EMR region had 2.67 (95% CI 1.10, 6.49) times the odds of cannabis use for non-binary participants compared to the EUR region.

### Substance use changes before and during COVID-19

Most transfeminine participants (*n* = 63, 52.5%) and most non-binary participants (*n* = 194, 55.4%) reported an increase in use of at least one substance since the start of the COVID-19 pandemic (Table 4). In comparison, the majority of transmasculine participants reported no increases (*n* = 20, 51.3%). Roughly half of those who used tobacco (*n*/*N* = 254/509) reported no change; 32.3% (*n* = 163) reported an increase since the start of COVID-19. Across all gender identities, more transmasculine participants reported an increase in alcohol use (*n* = 15, 21.1%) compared to transfeminine participants (*n* = 29, 13.2%) and non-binary participants (*n* = 97, 16.4%).

**Table 4:** Descriptive statistics of changes in substance use since the COVID-19 pandemic among transgender participants

	Transfeminine *n* (%)	Non-binary *n* (%)	Transmasculine *n* (%)	*P* value
Change in at least one substance used since COVID-19				0.63
Individuals responding to at least one substance change question	*n* = 120	*n* = 350	*n* = *39*	
Increase in at least one	63 (52.5%)	194 (55.4%)	19 (48.7%)	
No increase in at least one	57 (47.5%)	156 (44.6%)	20 (51.3%)	
Change in tobacco use since COVID-19				0.92
Decreased	18 (14.3%)	39 (12.3%)	5 (13.9%)	
No change	69 (54.8%)	167 (52.7%)	18 (50.0%)	
Increased	39 (31.0%)	111 (35.0%)	13 (36.1%)	
Change in alcohol use since COVID-19				0.52
Decreased	58 (26.4%)	162 (27.5%)	17 (23.9%)	
No change	133 (60.5%)	331 (56.1%)	39 (54.9%)	
Increased	29 (13.2%)	97 (16.4%)	15 (21.1%)	
Change in cannabis use since COVID-19				0.42
Decrease	6 (24.0%)	19 (23.2%)	3 (33.3%)	
No change	7 (28.0%)	38 (46.3%)	3 (33.3%)	
Increased	12 (48.0%)	25 (30.4%)	3 (33.3%)	

Due to the survey skip pattern, only individuals who reported prior substance use in the last 6 months were asked questions about change in substance use. Therefore, it was not possible to conduct multiple imputation by chained equation on samples to account for missingness. *COVID-19* novel coronavirus disease 2019

Chi-squared tests were conducted for categories with ≥ 5 respondents, while Fisher’s exact tests were conducted for categories with < 5 respondents (transmasculine participants reporting a change in cannabis use)

There were fewer responses to cannabis questions compared to tobacco or alcohol use questions, and therefore, it is difficult to identify trends. The plurality of transfeminine participants reported increased use of cannabis (48.0%, *n* = 12 of 25 responses). The plurality of non-binary participants reported no change in cannabis (46.3%, *n* = 38 of 82 responses). Transmasculine participants had high missingness for these questions, and it was not possible to discern descriptive trends.

## Discussion

We examined differences in substance use patterns and changes in the frequency of substance use during the COVID-19 pandemic by gender identity among a global TNB sample. Our cross-sectional study found the majority of TNB participants used tobacco and alcohol, and a notable minority used cannabis. Compared to transfeminine participants, alcohol use among transmasculine participants, and cannabis use among non-binary participants increased during the COVID-19 pandemic. We also presented the cross-sectional prevalence of reported changes, providing important estimates for clinical providers and health researchers working with TNB populations. Our study importantly reports differences in substance use patterns and the pandemic’s impact on substance use between TNB sub-groups, which has implications for designing substance interventions for these populations.

Our study is novel in reporting on a large sample of TNB participants, the majority of whom identified as non-binary. A recent synthesis of the prevalence and correlated factors for substance use among TNB participants noted that no study to date has drawn such distinctions, particularly around non-binary, transfeminine, and transmasculine participants [22]. Non-binary participants are often underrepresented in research, especially in substance use studies [22, 30, 52, 53]. Additionally, many substance use studies, including those conducted during the pandemic, have aggregated transgender participants with men who have sex with men, despite evidence that these populations have different needs [29, 30, 54]. Drawing distinctions between TNB subgroups is imperative in identifying the issues facing these individual subcommunities within the broader gender non-conforming community, to help tailor future research and interventions around substance use disorders and behavioral health for this population. The necessity of disaggregation also extends to understanding the impact of minority stress on TNB communities [33, 40, 55]. Experiences of both explicit and implicit discrimination and prejudice [55], as well as more ambient stress would likely lead to intra-group differences among TNB communities for how they may use substances to cope or socially engage with peers.

During the COVID-19 pandemic, compared to before the pandemic, transmasculine people reported the highest proportion of increased alcohol use in descriptive statistics, though we lacked power to determine statistical significance. Additionally, transmasculine participants were more likely to self-report lower income and educational attainment, factors often correlated with increased alcohol consumption and alcohol-related deaths among a general population [56–58]. Other studies have indicated that transmasculine individuals have higher rates of alcohol use compared to transfeminine individuals [38, 55]. Therefore, while our finding was not significant after adjustment, this line of research deserves future investigation to disentangle these effects and what factors contribute to alcohol use in this group. Health programs should also consider tailoring alcohol services to the unique needs of transmasculine communities to address external risk factors and internal beliefs related to alcohol use.

Transfeminine participants were less likely to use cannabis compared to non-binary participants, but those reporting cannabis use were more likely to report an increase during the pandemic compared to non-binary and transmasculine participants. We lacked power to discern the role of additional covariates, but potentially, it could be related to enacted stigma, such as violence and discrimination. Nuttbrock et al. (2014) found that changes in experiencing gender-based violence were associated with increased cannabis use, as well as other recreational substances, among transgender women [59]. Based on a recent proposed framework of minority stress among TNB groups [60], this would be related to the differential stressors they face as a marginalized group specific to their positionality as transgender women, compared to cisgender women or transgender men. Prior evidence similarly supports a differential effect of discrimination and anticipated stigma among TNB groups [61], and it is worth noting that transfeminine participants endorsed higher proportions of discrimination compared to non-binary and transmasculine participants in our sample. Our study did not examine specific experiences of transgender-related discrimination, and more research is needed to determine the role it may play on cannabis use.

Participants across all gender identities who used cannabis used the same amount or more since the start of the COVID-19 crisis. This is in contrast to a recent community-based study in Argentina, which found that substance use generally declined among TNB people during COVID-19 [62]. Given our study drew upon a global sample of participants, substance availability and pandemic-related factors that impact substance use likely differed from this Argentinian population, impacted by a wider range of socioenvironmental factors compared to a country-specific sample. Additionally, recruitment via a social networking and dating app, where individuals may be more connected to social scenes where substance use is common, may not mirror a community-based sample. Rather, the pandemic may have exacerbated existing stressors and created new ones, resulting in stress-coping through increased cannabis use. For example, LGBTQ + populations may have sheltered in place within households that were not gender affirming or faced disconnection from affirming communities and social networks [63]. Given cannabis use has been previously associated with anxiety disorders [64], and TNB people have higher prevalence of anxiety disorders compared to cisgender people [65, 66], the pandemic may have exacerbated prior stress-related disorders and substance use. Further research should directly investigate this.

### Limitations

Our study is not without limitations. The cross-sectional nature of the data limits assessing causality but likely aided recruitment for a large analytic sample, and our findings describe the prevalence of substance use overall, rather than substance use disorders. Our sample was limited to countries and participants who were able to access the app, and it is notable that access may have been limited due to country policies and hostile climates for LGBTQ + people. Our sample similarly may not generalize to all TNB populations, such as communities with lower education rates or who do not use dating apps. Another limitation is the use of self-report data, which may be subject to recall and response bias. Due to social desirability bias, participants may under-report their substance use and, in such a case, our findings would be biased towards the null hypotheses. To limit these potential biases, we asked participants about substance use changes in the last 6 months, as opposed to asking them to accurately recall the entire pandemic. Our data was also limited to any/none use, as questions with further granularity were not asked among participants. This was done given the length of the overall survey, which included nearly 60 questions and covered numerous important topics. Additionally, given the global sample, we controlled for geographic region to mitigate confounding by geographic differences; however, there may be residual confounding compared to controlling for geography at a more granular level. To compensate for this, we also used urbanicity in our models to add nuance in place-based factors. Future studies should directly investigate other regional norms that may have been involved in changing substance use patterns during the COVID-19 pandemic, such as changes in socializing in cities compared to socializing in rural areas [67]. Finally, we had high missingness on variables related to changes in substance use behaviors during the pandemic and we were not able to generate valid inferences. Samples with more tailored recruitment that can better reach substance-using TNB populations should be considered for future studies.

## Conclusions

Our study highlights the importance of considering intragroup differences to understand substance use patterns among transgender people, given that differences in the impact of lived experiences and risk factors on substance use behavior may vary by gender. Consistent with other studies, this global sample of transgender people found high levels of substance use at the onset of the pandemic. Our study demonstrated increases in cannabis use among transfeminine individuals. Health programming should consider focusing resources on TNB clients and taking a gender-specific, substance-specific approach to improve the health of these marginalized populations.

## Supplementary Information

**Additional file 1: Fig. S1.** Flowchart of participant inclusion in analytic sample from baseline study population in the Global COVID-19 Disparities Survey from October to November 2020. **Fig. S2.** Directed acyclic graph of risk factors for substance use among transgender and non-binary participants in the Global COVID-19 Disparities Survey from October to November 2020.

## Data Availability

The datasets generated and/or analyzed during the current study are not publicly available in order to maintain confidentiality agreements and the privacy of participants. Reasonable requests for anonymized data will be considered by the study administrators. Interested parties can contact the corresponding author for more information, HGD garrisondesany@jhu.edu.

## Abbreviations

AFR: African region
AMR: Region of the Americas
aOR: Adjusted odds ratio
CI: Confidence interval
COVID-19: Coronavirus disease of 2019
DAG: Directed acyclic graph
EMR: Eastern Mediterranean Region
EUR: European region
HIV: Human immunodeficiency virus
IQR: Interquartile range
LGBTQ: Lesbian, gay, bisexual, transgender, and queer
OR: Odds ratio
SEAR: Southeast Asian Region
TNB: Transgender and non-binary
WHO: World Health Organization
WPR: Western Pacific Region
