# Supplementary material for: Substance use patterns among a global sample of transgender and non-binary people during the COVID-19 pandemic
Source: BMC Glob Public Health. 2023 Sep 18;1:16. doi: 10.1186/s44263-023-00014-5 (PMC11622945; doi:10.1186/s44263-023-00014-5)
Supplement: Supplementary file 1 — Additional file 1: Fig. S1. Flowchart of participant inclusion in analytic sample from baseline study population in the Global COVID-19 Disparities Survey from October to November 2020. Fig. S2. Directed acyclic graph of risk factors for substance use among transgender and non-binary participants in the Global COVID-19 Disparities Survey from October to November 2020. [file 44263_2023_14_MOESM1_ESM.docx]

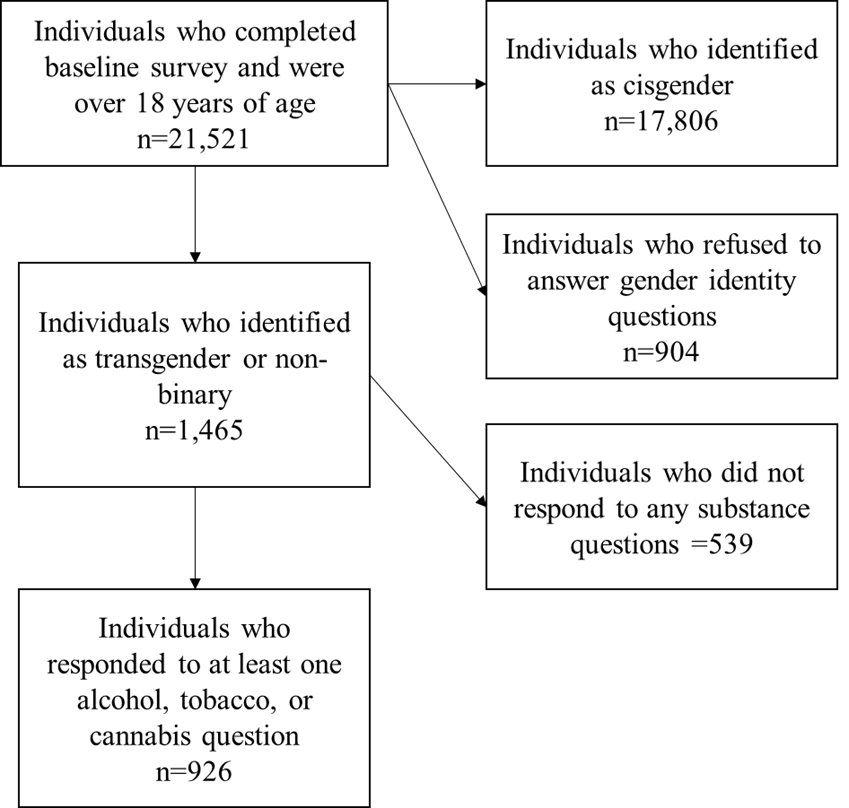


Fig. S1. Flowchart of participant inclusion in analytic sample from baseline study population in the Global COVID-19 Disparities Survey from October to November 2020.

Fig. S2. Directed acyclic graph of risk factors for substance use among transgender and non-binary participants in the Global COVID-19 Disparities Survey from October to November 2020.
